# Genomes, neurotoxins and biology of *Clostridium botulinum* Group I and Group II

**DOI:** 10.1016/j.resmic.2014.10.010

**Published:** 2015-05

**Authors:** Andrew T. Carter, Michael W. Peck

**Affiliations:** Institute of Food Research, Norwich Research Park, Colney, Norwich, NR4 7UA, UK

**Keywords:** *Clostridium botulinum*, Botulism, Botulinum neurotoxin, Genomics, Neurotoxin gene cluster

## Abstract

Recent developments in whole genome sequencing have made a substantial contribution to understanding the genomes, neurotoxins and biology of *Clostridium botulinum* Group I (proteolytic *C. botulinum*) and *C. botulinum* Group II (non-proteolytic *C. botulinum*). Two different approaches are used to study genomics in these bacteria; comparative whole genome microarrays and direct comparison of complete genome DNA sequences. The properties of the different types of neurotoxin formed, and different neurotoxin gene clusters found in *C. botulinum* Groups I and II are explored. Specific examples of botulinum neurotoxin genes are chosen for an in-depth discussion of neurotoxin gene evolution. The most recent cases of foodborne botulism are summarised.

## 1 Introduction

### 1.1 General properties of *Clostridium botulinum*

*C. botulinum* is, unusually for a bacterial species, defined by a single property that is the ability to produce botulinum neurotoxin. Four discrete groups of bacteria are recognised within *C. botulinum*. *C. botulinum* Groups I and II are primarily responsible for human botulism, *C. botulinum* Group III is responsible for botulism in various animal species, and *C. botulinum* Group IV does not appear to be associated with botulism in humans or animals [1–3]. *C. botulinum* Group I (proteolytic *C. botulinum*) is a highly proteolytic bacterium that can also degrade a range of carbohydrates (Table 1, [4–6]). It is a mesophile, with minimum and optimum growth temperatures of 12 °C and 37 °C, respectively (Table 1). Spores formed by strains of *C. botulinum* Group I are highly heat resistant, and the “Botulinum cook” (121 °C/3 min) given to low acid canned foods is designed to inactivate these spores. The number of neurotoxin genes present in the genome, and neurotoxins actually formed by strains of *C. botulinum* Group I is variable, with strains possessing from one to three neurotoxin genes, and forming one to three different neurotoxins [7,8]. Strains that possess two neurotoxin genes may form either one active toxin (e.g. type A(B) strains form active type A neurotoxin, but not active type B neurotoxin), or two active toxins (e.g. type Ab strains form more type A neurotoxin than type B neurotoxin). *Clostridium sporogenes* is often viewed as a non-toxigenic equivalent of *C. botulinum* Group I (Table 1). *C. botulinum* Group II (non-proteolytic *C. botulinum*) is a highly saccharolytic bacterium that ferments a range of carbohydrates (Table 1). It is a psychrotroph, with minimum and optimum growth temperatures of 3.0 °C and 30 °C, respectively (Table 1). Spores formed by strains of *C. botulinum* Group II are of moderate heat resistance (Table 1). Until recently it was understood that all strains of *C. botulinum* Group II possessed a single neurotoxin gene and formed a single neurotoxin, however it was recently discovered that strains of *C. botulinum* Group II type F also contain a fragment of a type B and a type E neurotoxin gene [9]. Bacteria are described that resemble *C. botulinum* Group II, but do not form neurotoxin; however they have not yet been given a formal name.

### 1.2 General properties of the botulinum neurotoxins

There are seven confirmed major botulinum neurotoxins (types A–G, with type H recently reported but yet to be verified [10,11]) and more than thirty different subtypes [12]. Botulinum neurotoxins are the most potent naturally-occurring substance, with as little as 30–100 ng (3000 MLD_50_ (mouse minimum lethal doses)) of neurotoxin sufficient to cause human botulism. This estimate is based on the amount of neurotoxin consumed in cases of foodborne botulism and from various animal experiments [13–15]. Botulinum neurotoxins are 150 kDa proteins that comprise a heavy chain (100 kDa) and a light chain (50 kDa). The heavy chains have two functional domains. The C-terminal domain is involved in neurotoxin binding to the nerve cell, with the N-terminal domain involved in movement of the light chain into the cytoplasm of the nerve cell. The light chains are zinc endopeptidases that are active within the nerve cell. Here they selectively cleave proteins (e.g. SNAP-25, VAMP) of the neurotransmitter-vesicle docking/fusion complex, destabilising this complex. This prevents neurotransmitter release and leads to a flaccid muscle paralysis [16,17]. *C. botulinum* Group I (proteolytic) strains form neurotoxins only of type A, B, F and H; strains of *C. botulinum* Group II (non-proteolytic) form neurotoxins of type B, E or F (Table 1).

**Fig. 1:**
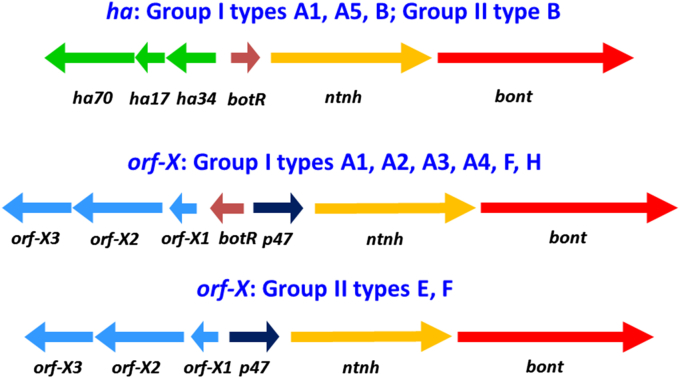
Two major neurotoxin cluster arrangements in *C. botulinum* Group I and *C. botulinum* Group II [11]. Note that the *botR* gene, the product of which acts as a positive regulator for expression of the neurotoxin gene cluster, is not present in *C. botulinum* Group II *orf-X* neurotoxin gene clusters.

The botulinum neurotoxins form complexes with accessory proteins (e.g. haemagglutinin (HA) and non-toxic-non-haemagglutinin (NTNH)) of various sizes from 300 kDa to 900 kDa. These accessory proteins protect the neurotoxin and may facilitate its absorption into the body. Indeed a recent publication has shown that the haemagglutinin complex of the type A neurotoxin specifically binds the cell adhesion protein, E-cadherin, compromising the tight junction between gut epithelial cells to facilitate absorption of the neurotoxin complex from the gut lumen into the body [18]. Genes encoding the neurotoxins and accessory proteins are situated together in one of two conserved neurotoxin clusters (*ha* cluster and *orf-X* cluster), both of which comprise two transcriptional units (Fig. 1). For *C. botulinum* Groups I and II, these are located on the chromosome or a plasmid [5]. One transcriptional unit in the *ha* cluster encodes genes for the neurotoxin and NTNH, while a second transcriptional unit (transcribed in the opposite direction) encodes three HA genes (Fig. 1). A gene encoding a positive regulator (sigma 70 factor) of the *ha* cluster is located between these two transcriptional units [19]. The *orf-X* cluster includes a transcriptional unit encoding genes for the neurotoxin and NTNH (as with the *ha* cluster). The second transcriptional unit contains a group of three open reading frames of unknown function (*orf-X1*, *orf-X2*, *orf-X3*) and lacks the three HA encoding genes. The *orf-X* cluster contains a gene (*p47*) of unknown function; interestingly, the gene encoding the positive regulatory protein (*botR*) also appears to be present in the *orf-X* neurotoxin gene cluster in strains of *C. botulinum* Group I, but not in those of *C. botulinum* Group II (Fig. 1). Other differences between otherwise closely related neurotoxin gene clusters have been noted, which are presumably indicators of past DNA recombination events. For instance, intergenic distances within the neurotoxin gene cluster may vary; some type A1 strains with an *orf-X* cluster possess an extra 0.6 kb between the *orf-X2* and *orf-X3* genes, and the Ba4 strain, CDC 657, shares the feature of an extra 1.2 kb between the *orf-X1* and *botR (p21)* genes with the type A3 strains [20]. More subtly, the promoter region of the *botR* gene of type A5 strains lacks a 76 nucleotide (nt) region which encompasses the sigma factor binding sites thought to be required for autoregulation of BotR [21,22].

### 1.3 Foodborne botulism

Botulism is a severe disease of humans and animals, with a high fatality rate (ca. 5–10% of cases). Typical symptoms are flaccid muscle paralysis; often initially blurred vision, followed by an acute symmetrical descending bilateral paralysis, and if not treated, ultimately paralysis of the respiratory or cardiac muscles. If severe cases are not fatal, then full recovery may take months or even years. There are three major types of botulism in humans; infant/intestinal (adult) botulism, wound botulism and foodborne botulism. Infant/intestinal (adult) botulism is an infection associated with cell multiplication and neurotoxin formation in the gut, and wound botulism is an infection associated with cell multiplication and neurotoxin formation in a wound (often following drug abuse). Foodborne botulism is an intoxication caused by consumption of neurotoxin pre-formed in the food. The successful prevention of foodborne botulism depends on (i) identifying appropriate control measures when new processing technologies are introduced or modified, and (ii) ensuring that these effective control measures are applied correctly. This requires the control of two physiologically distinct bacteria (*C. botulinum* Groups I and II, see Table 1). The commercial implications of foodborne botulism can be very severe. For example, in New Zealand in 2013, a milk product was recalled that was not associated with any cases of botulism, but suspected of being contaminated with *C. botulinum* (ultimately found to be free of contamination), and according to newspaper reports cost hundreds of millions of dollars.

The failure to effectively apply the botulinum cook (121 °C/3 min) to canned or bottled foods has led to many outbreaks of foodborne botulism associated with *C. botulinum* Group I [23–51](Table 2). For example, a large outbreak in Thailand in 2006 (209 cases) was associated with consumption of inadequately home-canned bamboo shoots [33,34]. Inadequate thermal processing of cans of a commercial hot dog chilli sauce in 2007 in USA was associated with eight botulism cases, and initially led to the recall of 39 million cans, then an expanded recall of 111 million cans [39]. The canning facility was eventually closed. Temperature abuse of foods intended to be stored chilled has also been responsible for several severe outbreaks of foodborne botulism, including those associated with commercial chilled carrot juice and commercial chicken enchiladas (Table 2). A number of recent outbreaks of foodborne botulism in the USA have been associated with illicit prison alcohol (”pruno”) (Table 2).

Strains of *C. botulinum* Group II forming type B neurotoxin are often associated with foodborne botulism and meat products in Europe, and sometimes with fish products in North America [52–57]. It is likely that the first strain of *C. botulinum* isolated by van Ermengem in 1895, following an outbreak of foodborne botulism in Belgium associated with salted ham, was a strain of *C. botulinum* Group II type B [58]. Recent foodborne botulism outbreaks in Iceland (at least one associated with blood sausages), UK (jar of home-preserved pork from Poland), and France (home-prepared ham) were also associated with *C. botulinum* Group II type B [44,57]. Botulism outbreaks involving *C. botulinum* Group II type E have been most frequently associated with fish (e.g. vacuum, smoked, salted, dried), and home-prepared foods in the north of Canada and Alaska (e.g. fermented beaver tail and paw, “muktuk”) (Table 2). For example, a botulism outbreak in France in 2009 (3 cases) was associated with consumption of commercial vacuum-packed hot-smoked whitefish in which *C. botulinum* Group II type E had grown and formed neurotoxin, probably during temperature abuse (Table 2). Minimally heated, chilled foods are a current concern [5,6,59,60].

### 1.4 Whole genome sequencing and *C. botulinum*

In 2007, the first genome of a strain of *C. botulinum* was published (Group I strain ATCC 3502) [61]. This article provided the first insights into the genome of a strain of *C. botulinum*, and heralded the start of a new era of *C. botulinum* genomics. The sequence of a number of *C. botulinum* genomes is now available in public databases. The cost of genome sequencing has fallen by many orders of magnitude over recent years, and a number of large genome sequencing projects are presently in progress. These will extend our understanding of the biology of the organisms that are *C. botulinum*. Our present knowledge of the genomics of *C. botulinum* Groups I and II is detailed below. The sequencing of botulinum neurotoxin genes and their associated clusters started more than twenty years ago, but has been considerably accelerated by advances in whole genome sequencing. This resulted in an appreciation of the variability of botulinum neurotoxin genes and their associated gene clusters. Current understanding of neurotoxins formed by strains of *C. botulinum* Groups I and II is described in this review.

## 2 Genomics of *C. botulinum* Groups I and II

### 2.1 Comparison of whole genome sequences

The first genome to be published was that of *C. botulinum* Group I strain ATCC 3502 [61]. The genome comprised a chromosome (3.89 Mb) and a small plasmid (16.3 kb) that encoded a bacteriocin. The genome had a low G + C content (28.2%). Other strains of *C. botulinum* Group I that have been subsequently sequenced have been found to be highly similar. The genomes of strains of *C. botulinum* Group II are of a similar size, and have a marginally lower G + C content (27.5%) [62].

**Fig. 2:**
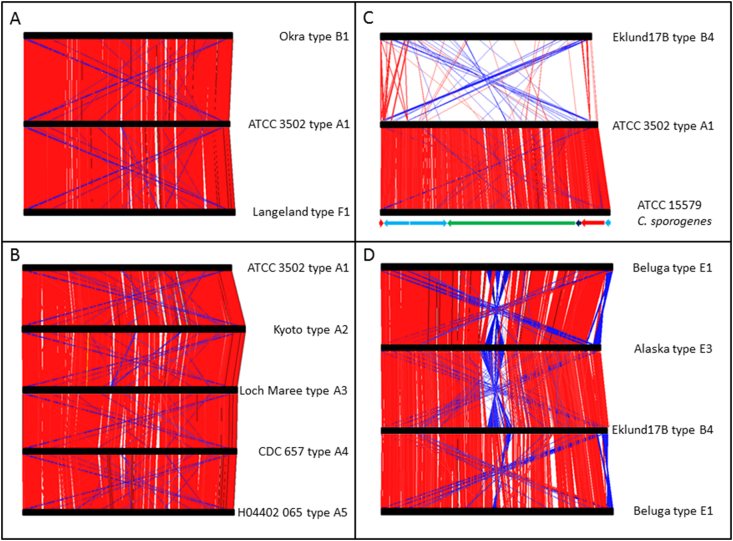
ACT comparisons of *C. botulinum* genomes. Red blocks indicate DNA homology (>90%) between paired genomes. Circular genomes are represented as linear horizontal black bars, with the gene annotated as coming first after each origin of replication positioned at the left hand end of each bar. A: *C. botulinum* Group I genomes of strains forming neurotoxins types A, B and F. B: *C. botulinum* Group I genomes of strains forming neurotoxin subtypes A1 – A5. C: Genomes of the *C. botulinum* Group I strain ATCC 3502, the *C. botulinum* Group II strain Eklund 17B, and the non-neurotoxigenic *C. sporogenes* ATCC 15579. Note that the contigs available in GenBank for the unfinished genome of *C. sporogenes* ATCC 15579 have been manually edited to generate a ‘best fit’ genome. The arrows below the black bar representing the genome of ATCC 15579 are coloured according to contig number: red, contig 488 (GenBank accession number ABKW02000002); light blue, contig 478 (ABKW02000003); green, contig 493 (ABKW02000004); dark blue, contig 486 (ABKW02000001). The arrows also indicate whether the contigs have been reverse/complemented in order to respect the genome of ATCC 3502, and that two contigs (478 and 488) have been broken apart, also in order to match this genome. D: Genomes of the *C. botulinum* Group II strains Beluga, Alaska and Eklund 17B. (For interpretation of the references to colour in this figure legend, the reader is referred to the web version of this article.)

The genomes of strains of *C. botulinum* Group I that form type A, B, or F (but not type H) neurotoxin are presently available for analysis. Three commonly studied strains of *C. botulinum* Group I are ATCC 3502 (type A1), Okra (type B1) and Langeland (type F1). The genomes of these three representative strains have been compared using the Artemis Comparison Tool (ACT, [63]), a software programme freely available from the Wellcome Trust Sanger Institute website. Fig. 2A shows the results of this comparison, with ATCC 3502 in the centre, Okra at the top, and Langeland at the bottom. The red blocks in the graphic which connect each genome (represented as a black horizontal bar) depict regions of high (>90%) homology. It is apparent that although forming serologically distinct toxin types, these *C. botulinum* Group I genomes are closely related, displaying relatively minor insertions/deletions of genetic information with respect to each other.

Within each neurotoxin type, with the exception of G, a number of subtypes have been identified. For example, the genomes of strains forming five type A subtypes (termed A1, A2, A3, A4, and A5) are presently available [12,64]. Fig. 2B is an ACT comparison of the genomes of strains which each form one of the subtypes A1-A5. As before, although clear differences in DNA identity can be observed, these are confined to relatively small regions, spanning up to 90 genes (the largest white gap in the comparison between the A3 and A4 strains in Fig. 2B). The level of sequence identity is very similar to that seen in Fig. 2A, thus at this level of analysis, strain ATCC 3502 (type A1) does not appear to be more closely related to the strains forming other type A neurotoxins than to strains forming type B or F neurotoxins. Similar observations were also made using a DNA microarray based on the genome of strain ATCC 3502, with the microarray analysis also revealing more subtle differences (section 2.2). It is, however, apparent that all of these strains of *C. botulinum* Group I exhibit synteny.

Once we stray from the boundaries defined by membership of *C. botulinum* Group I, DNA sequence homology rapidly declines. Fig. 2C demonstrates this quite dramatically with a comparison between the genomes of *C. botulinum* Group I ATCC 3502, and *C. botulinum* Group II Eklund 17B. The ACT comparison software struggles to find enough homology to assign any red blocks at all. In contrast, although designated as a different species by virtue of its failure to form botulinum neurotoxin, *C. sporogenes* ATCC 15579 shares enough identity with ATCC 3502 to confuse a taxonomist who was unaware of its toxin status. The ACT analyses presented here confirm the results of a previously described microarray study, where an array based on the genome of ATCC 3502 was successfully used to place strains of *C. sporogenes* within a meaningful family tree, but completely failed when used with DNA from Eklund 17B [65].

At present, fewer complete genomes from *C. botulinum* Group II are available for study. However, comparison of the genomes reveals clear regions of high homology, but the gaps in between the red blocks seem to be larger and more frequent than for *C. botulinum* Group I (Fig. 2D). This suggests that *C. botulinum* Group II is more diverse than *C. botulinum* Group I, confirming earlier studies that used alternative approaches [15]. In addition, the blue blocks, which represent DNA sequence inversions with respect to the genome with which it is compared, are also more obvious. Interestingly, the genome of strain Alaska, which forms neurotoxin type E3, carries a large region at its centre (in this linear depiction) which is inverted with respect to the genome of Beluga (type E1) and of Eklund 17B (type B4). The genome of Beluga was used twice in this instance (top and bottom) to demonstrate that the inversion is indeed in the Alaska genome, as Beluga aligns quite well with Eklund 17B (Fig. 2D). The significance of this apparent inversion in the Alaska genome is not known at present.

### 2.2 Comparison using DNA microarrays

**Fig. 3:**
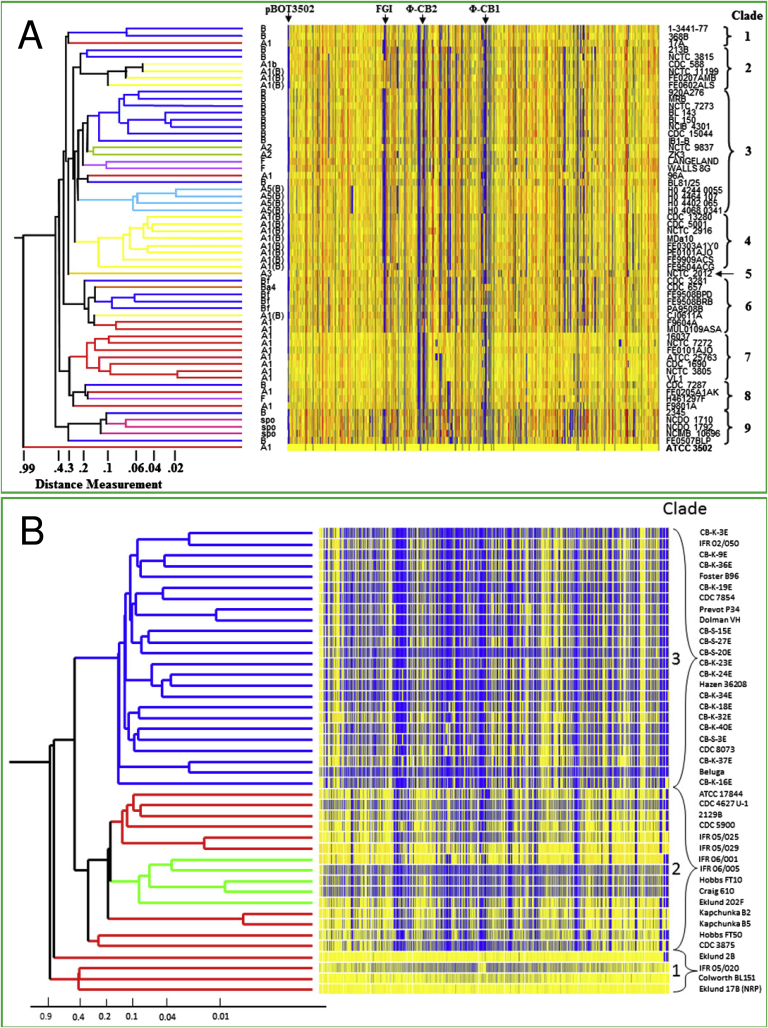
Heatmaps and dendrograms generated by two colour microarray analysis of genomic DNA from strains of *C. botulinum* Group I (panel A) and of *C. botulinum* Group II (panel B). Microarray probes for the *C. botulinum* Group I analysis were derived from the genome sequence of ATCC 3502 [61,65] and for the *C. botulinum* Group II analysis from the genome sequence of Eklund 17B [62]. Competitive hybridisations for the *C. botulinum* Group I analyses were performed by mixing genomic DNA of strain ATCC 3502 with that of the test strain, each DNA having been labelled with a different fluorescent dye, before adding to the microarray. Similarly, labelled DNA from strain Eklund 17B was used as the hybridisation reference for the *C. botulinum* Group II experiments. In each heatmap, a yellow colour signals that the test strain genome shares >85% homology with a gene probe on the microarray, generally implying that a very similar gene may be present (with the caveat that due to the small size (60 nt) of each microarray probe, false signals may be generated by small sequence differences, giving a level of background ‘noise’ which has to be normalised during data processing). The bottom, horizontal lane of each heatmap is an internal control experiment, and represents the result of hybridising the reference strain DNA for each Group with itself; any bars which lack a yellow colour in these two lanes indicate the position of microarray probes which for technical reasons have failed to hybridise to their cognate DNA sequence. The Group I clades (panel A) do not respect neurotoxin types formed, while the Group II clades (panel B) do respect neurotoxin types formed; i.e. clade 3 = type E, clade 2 = type B or type F, clade 1 = type B strains most closely related to Eklund 17B (hence the greater proportion of yellow bars in these heatmaps). (For interpretation of the references to colour in this figure legend, the reader is referred to the web version of this article.)

DNA microarrays provide an alternative to whole genome sequencing for analysis of whole genomes; they are particularly useful for comparison of many closely related genomes, using a well-characterised, sequenced strain as a hybridisation reference. Fig. 3 displays the main set of results from two different studies, in which a DNA microarray was used to compare sequences representing all predicted genes of the sequenced *C. botulinum* Group I strain ATCC 3502 with the unknown ones of several strains of the same Group (Fig. 3A), and similarly all predicted genes of the Eklund 17B genome were compared with unknown ones from other members of *C. botulinum* Group II (Fig. 3B). Strains tested using the *C. botulinum* Group I microarray included those forming type A, A(B), B, Bf and F neurotoxins, plus several examples of the closely related but non-toxigenic *C. sporogenes*. Strains tested using the *C. botulinum* Group II microarray included those forming type B, type E or type F neurotoxin [62,65].

When presented side-by-side for the first time, the similarities and differences between *C. botulinum* Groups I and II, as demonstrated by ACT analysis (Fig. 2), become readily apparent. The greater quantity of yellow colour in the heatmap for *C. botulinum* Group I than for *C. botulinum* Group II further confirms that the genetic variation between members of *C. botulinum* Group I is much less than that apparent for members of *C. botulinum* Group II (Fig. 3). Furthermore, the coloured lines at the left of each dendrogram in Fig. 3 indicate the type of neurotoxin formed by the tested strain. In the case of *C. botulinum* Group I, although some groupings are clearly noticeable, the clustering of strains according to the majority of the genetic information contained on the chromosome (i.e. clades on the right hand side of Fig. 3A) does not always align according to the type of neurotoxin formed (Fig. 3A). Interestingly, strains of *C. sporogenes* fitted well with the *C. botulinum* Group I strains [65]. That said, a recent genetic and functional study of spore germination receptors showed there to be significant physiological differences between *C. sporogenes* and *C. botulinum* [66]. The position with *C. botulinum* Group II is, however, very different (Fig. 3B). *C. botulinum* Group II can be relatively easily organised into only three clades, mostly true to their neurotoxin type, suggesting that horizontal transfer of neurotoxin gene clusters occurs less frequently in *C. botulinum* Group II than in *C. botulinum* Group I. This is particularly striking for strains which form type E neurotoxin, which group together in a clade that is obviously genetically well-separated from those that form type B or type F neurotoxin. This genetic homogeneity may be due to a better defence strategy against invading foreign DNA, less overall recombinogenicity of DNA, or may simply reflect the geographical isolation of clades within *C. botulinum* Group II.

Comparison of the large, seemingly unbroken blocks of synteny shared by the B and E serotypes that are displayed in the ACT figures, and the number of blocks coloured blue in the Group II microarray heatmap hints at an inconsistency between these analyses (Figs. 2 and 3). The reason for this apparent discrepancy is that very small sequence differences (often of only one or two nucleotides) were found to affect hybridisation of test DNA to the microarray 60-mer gene probes, generating false negatives; whereas the ACT analysis, based on an average sequence identity over a much greater portion of each chromosome, generated a ’positive’ DNA homology match at approximately 90% or greater. That said, the higher degree of false negative results from the microarray analysis did not seem to greatly influence the placement of each strain within its appropriate clade.

The apparent insertion of a cluster of *C. botulinum* Group II type F strains into the *C. botulinum* Group II type B clade (Fig. 3B, group of green-coloured tree branches surrounded by red-coloured ones) led to a more detailed investigation, using high density genome sequencing. This revealed evidence for an insertion of 34 kb of foreign DNA, containing the type F6 neurotoxin gene cluster, into a chromosome that is otherwise very closely related to that of a *C. botulinum* Group II type B4 [9]. Since all *C. botulinum* Group II type B4 strains so far examined carry their neurotoxin gene cluster on a plasmid, it is possible that loss or integration of parts of this plasmid may have represented one of the several evolutionary steps that would be required to produce the current type F6 genome [9].

Furthermore, the apparent lack of horizontal transfer of neurotoxin gene clusters in *C. botulinum* Group II, compared to that observed for *C. botulinum* Group I, contrasts with the frequency of occurrence of similar neurotoxin gene clusters in either a chromosomal or plasmid location in the latter (Tables 3 and 4). The fact that in all *C. botulinum* Group II type E and F strains, the neurotoxin gene cluster is inserted into a gene associated with DNA recombination, may imply that this represents an extra level of ‘genetic effort’ required to circumvent a normally very robust system to prevent integration of foreign DNA into the chromosome of *C. botulinum* Group II.

## 3 Neurotoxin gene clusters of *C. botulinum* Groups I and II

### 3.1 Location and mobility of neurotoxin gene clusters

The results from microarray analyses suggested that in *C. botulinum* Group I, neurotoxin gene clusters did not respect conventional vertical routes of heredity, but rather demonstrated an apparent ability to be acquired by horizontal transmission [65]. These observations added strength to the findings from early DNA sequencing experiments, which had revealed the presence of bacterial insertion sequence (IS) elements flanking the 5’- and 3’- boundaries of many neurotoxin gene clusters [61,67]. Insertion sequence elements (IS elements) are short sequences of DNA that encode transposase enzymes that promote their translocation and contribute to genetic recombination between bacteria; those found flanking *C. botulinum* neurotoxin gene clusters are usually non-functional and incomplete [68], but their presence suggested the possibility that they had been involved in horizontal acquisition of the neurotoxin gene cluster. The availability of genome sequences has made it possible, for the first time, to test this hypothesis by searching for signs of insertion of neurotoxin gene clusters into a previously undisturbed region of a bacterial chromosome. At present, three sites of chromosomal insertion, *oppA/brnQ*, *arsC* and *pulE* have been identified for *C. botulinum* Group I; and two, *rarA* and *topB* have been identified for *C. botulinum* Group II [7,9,69]. Table 3 summarises the present state of knowledge regarding the neurotoxin gene clusters of *C. botulinum* Group I, with respect to accessory gene status (i.e. *ha* or *orf-X*), whether the neurotoxin gene clusters occur singly or multiply within a genome, and if known, the chromosomal locus for each cluster. There are several examples where the neurotoxin gene cluster of a specific neurotoxin subtype can be found on a plasmid or on the chromosome [69,70](Table 3). ACT comparisons have demonstrated large regions of synteny between neurotoxin gene cluster-bearing plasmids of *C. botulinum* Group I, to the extent that it can be postulated that these too share preferred sites of insertion for the neurotoxin gene cluster [7,69]; however these have not yet been formalised by identification of flanking genes of known function. For *C. botulinum* Group II, the current position with respect to accessory gene status (i.e. *ha* or *orf-X*), and neurotoxin gene cluster location (plasmid or chromosomal locus) for each cluster is summarised (Table 4). Less is known about the neurotoxin gene clusters of *C. botulinum* Group II, as indicated by the relative sizes of Tables 3 and 4. At the time of writing, large-scale genome sequencing programmes are underway, including those for *C. botulinum* Group II, so this disparity may soon be addressed. Interestingly, horizontal transmission of neurotoxin gene clusters is less evident amongst members of *C. botulinum* Group II [9,62]. Until recently the only sequenced genome was that of strain Eklund 17B, which carries the type B4 neurotoxin gene cluster on a plasmid [62]. More recently, the sequences of ten more neurotoxin gene cluster-bearing plasmids of *C. botulinum* Group II type B4 have been published [57]. As with the plasmid of Eklund 17B [68], there is very little homology of these with any known plasmid from *C. botulinum* Group I; although these 11 type B4 plasmids share large regions of synteny amongst themselves and they represent a closely related cluster within *C. botulinum* Group II. The neurotoxin gene cluster of strains of *C. botulinum* Group II type E appears to be most frequently located on the chromosome, although PFGE analysis has recently revealed the presence of three strains of *C. botulinum* Group II type E1 that possess a neurotoxin gene cluster-bearing plasmid of about 146 kb [71]. The neurotoxin gene cluster of strains of *C. botulinum* Group II type F is chromosomally-located [9]. Further information on the type E1 plasmids and any others from *C. botulinum* Group II is needed before any meaningful analysis of preferred insertion site of the neurotoxin gene cluster within plasmids can be carried out.

### 3.2 Neurotoxin serotypes and neurotoxin gene subtypes

Serological methods were first used to distinguish botulinum neurotoxins more than a century ago. Initial work by Leuchs demonstrated that botulinum neurotoxins formed by two European strains of *C. botulinum* were antigenically distinct, with antitoxin raised against one neurotoxin not cross-neutralising neurotoxin formed by the other strain [72]. Later, Burke recognised two antigenically distinct botulinum neurotoxins, and designated these as types A and B [73]. This work established the use of serological methods based on type-specific antitoxin to distinguish botulinum neurotoxin serotypes, and has now led to the recognition of seven confirmed botulinum neurotoxin serotypes, types A–G, with a recently identified type H that remains to be verified [11,17]. However, it soon became obvious that there was also some variation within a single botulinum neurotoxin type.

The sequencing of botulinum neurotoxin genes and derivation of their amino acid sequence has rapidly advanced our understanding of the neurotoxins. It has been found that the amino acid differences between the seven neurotoxin serotypes (A to G) range from 37.2% to 69.6% [12]. Importantly, the classification of neurotoxin types based on the use of serological methods has been supported. Additionally, novel findings have been made on the variation within a single neurotoxin type with more than thirty neurotoxin subtypes described in the literature [12]. However, this has led to a debate on what might constitute a botulinum neurotoxin type or subtype. For example, should neurotoxin types continue to be defined on the basis of traditional serological methods or instead should they be based on the derived protein sequence? In a similar manner, how should a neurotoxin subtype be defined? We are probably moving towards the classification of botulinum neurotoxin types based on the derived protein sequence. The definition finally adopted needs to be an operational definition that is entirely consistent with the traditional serological approach. An advantage of a sequencing-based approach is that it is possible to compare neurotoxins formed by strains present in different laboratories; the transfer of strains between laboratories being heavily restricted by bioterrorism concerns.

In 2005, it was proposed that a *C. botulinum* neurotoxin gene might be defined as a subtype if it encoded a protein sequence that differed from the standard neurotoxin type by at least 2.6% [74]. Since then, many neurotoxin gene sequences have been determined, and some commonly accepted subtypes, for example type B3, differ from their closest relatives by less than 2.6% at the amino acid level [75]. Moreover, not all amino acid substitutions would have the same impact on the biological function of the neurotoxin, including its ability to be neutralised using the traditional antibody-based approaches. To this end, it has been proposed that newly discovered neurotoxin gene variants be named as such, until it has been established that they display different neurotoxin biology such as toxicity, antigenicity or substrate cleavage site [76]. The importance of the genetic background in which the neurotoxin gene is found has also been emphasised [20]. Certainly, the current position is ill-defined and inconsistent, and a rational approach is required within the botulinum community. As more and more neurotoxin gene sequences appear, boundaries have become blurred, and to highlight this we have reviewed the position with regard to *C. botulinum* Group I type A and its subtypes, and *C. botulinum* Groups I and II type B and its subtypes, as examples.

**Fig. 4:**
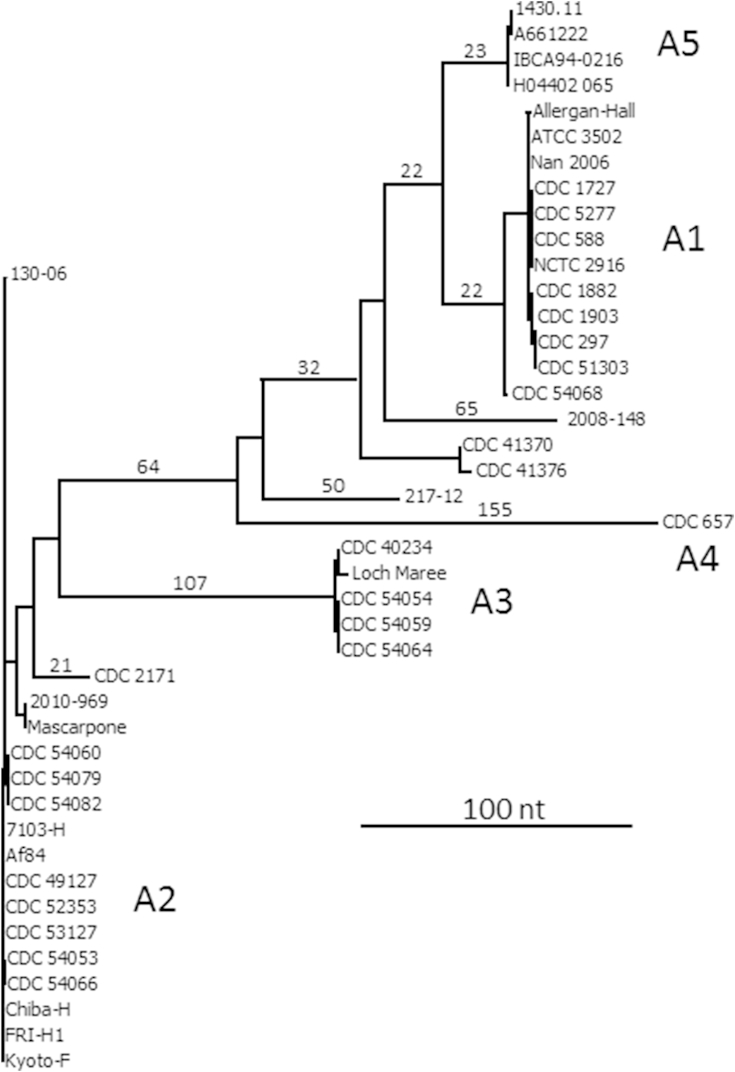
Dendrogram generated by pairwise comparison of the coding region of *C. botulinum* Group I neurotoxin gene subtypes A1-A5. Many examples available in GenBank that are identical to subtypes A1 and A2 depicted in this dendrogram have been omitted for clarity. Approximate values for the nucleotide differences used to generate the tree branch points are positioned above the major branches.

The neurotoxin gene sequence of five type A subtypes (A1 to A5) are available from GenBank. A dendrogram has been generated by pairwise comparison of these sequences (Fig. 4). Many more sequences are deposited, but are identical to those included here. As an indicator of scale, the main group of type A2 genes are within 2 nt of identity to each other; Mascarpone and CDC 2171 differ by 9 and 37 nt, respectively, to Kyoto-F. In the A3 group Loch Maree differs by 8 nt to the three CDC 540XX examples, which are identical to each other; Loch Maree differs by 143 nt to Kyoto-F and by 333 nt to the CDC 657 A4 gene. This gene in turn differs by 250 nt to the A5 genes, which differ by 53 nt to the A1 gene of ATCC 3502. Interestingly, the type A genes of CDC 41370, CDC 41376, 217-12, 2008-148 and CDC 54068 are placed separately, between the A1 and A4 branches, which indicates that some or all of them may represent a new subtype [67]; as an example, CDC 41376 differs by 249 nt to the A4 gene, and by 87 nt to the A1 gene of ATCC 3502. The A2 neurotoxin gene of CDC 2171 also displays sufficient diversity from the main body of type A2 genes to have been noted as another possible candidate for a new subtype [76]. Indeed a recent review has identified ten type A subtypes [17]. In summary, considering the genes encoding botulinum neurotoxin serotype A only, the DNA sequence identity of the different subtypes can vary from 2 nt to 333 nt.

**Fig. 5:**
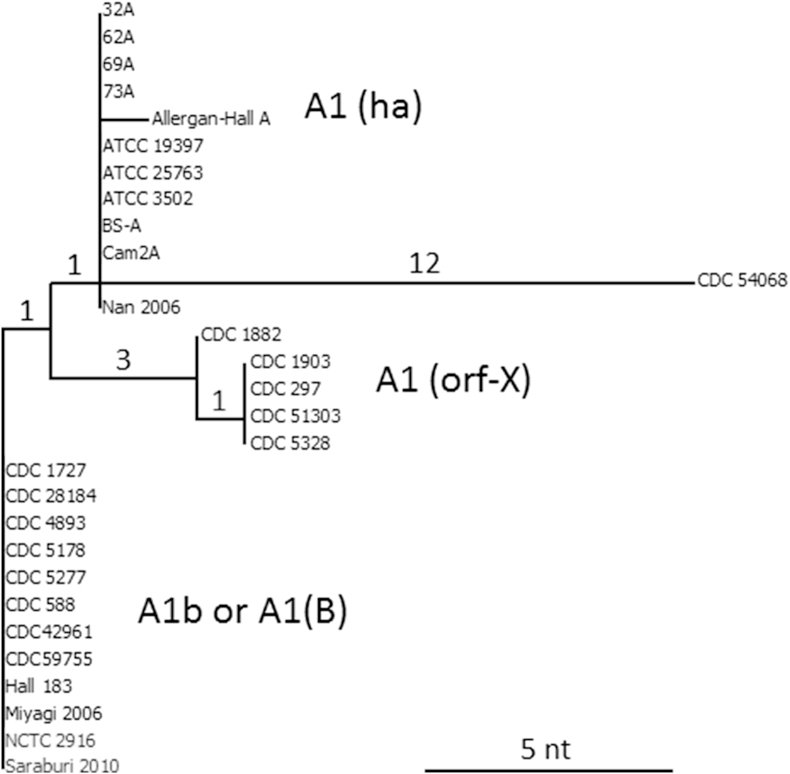
Dendrogram generated by comparison of neurotoxin genes of *C. botulinum* Group I type A1. Values for the nucleotide differences used to generate the tree branch points are positioned above the major branches.

Close examination of Fig. 4 hints at the presence of distinct sub-groups within the branch containing the type A1 genes. A similar pairwise alignment was performed using only type A1 gene sequences, generating a dendrogram (Fig. 5). Information for strains 73A, BS-A, Cam2A, CDC 42961 and CDC 59755 was derived from microarray data (GEO accession numbers GSM402914, GSM402916, GSM402932, GSM402918 and GSM402936, respectively). Information for CDC 54068 is limited to sequence of the neurotoxin gene only (GenBank JX127207). It can be seen that all type A1 genes are very closely related; using ATCC 3502 as an arbitrary standard, the A1 gene of Allergan-Hall A differs by only 1 nt; those of the NCTC 2916 cluster by 2 nt, those of the CDC 297 cluster by 5 nt and CDC 54068, which featured as an outlier in Fig. 5, by 12 nt. The three main groups are labelled according to the status of their *ha* or *orf-X* neurotoxin gene cluster, also whether the A1 gene is in a genetic background which includes a B gene (expressed or silent). Even though the number of single nucleotide polymorphisms (SNPs) separating the groups in this dendrogram is small, nevertheless there is a correlation between genetic background and type A1 cluster.

**Fig. 6:**
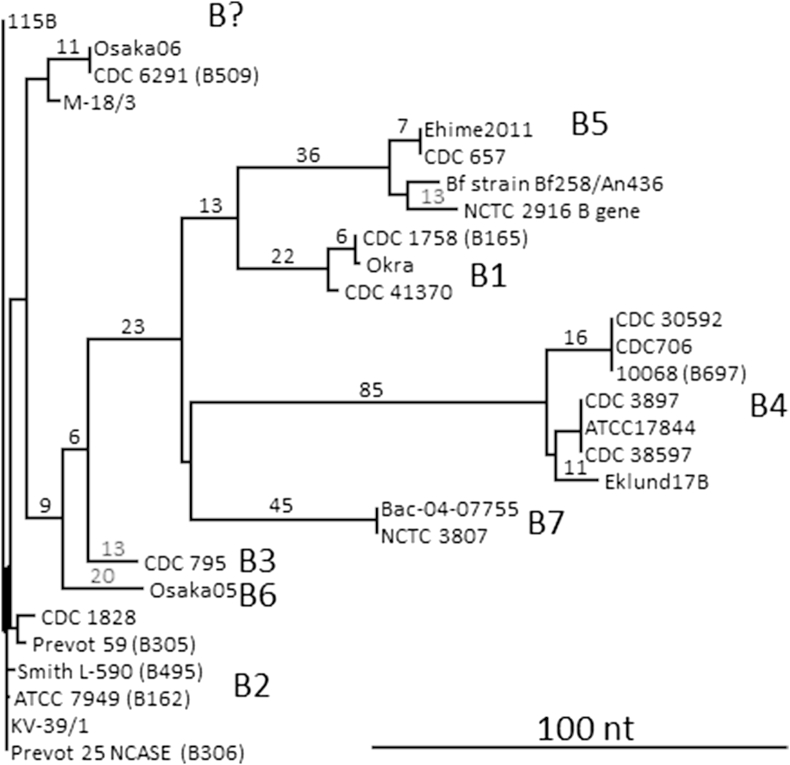
Dendrogram generated by pairwise comparison of the coding region of *C. botulinum* Group I and II type B genes. Approximate values for the nucleotide differences used to generate the tree branch points are positioned above the major branches.

Type B neurotoxin is formed by strains of *C. botulinum* Groups I and II, and the type B neurotoxin gene sequences are compared (Fig. 6). Considering the large genetic and physiological gap which separates *C. botulinum* Groups I and II, it is perhaps not surprising that each type B subtype appears to be exclusive to *C. botulinum* Group I or Group II. Subtype B4 appears to be only found in *C. botulinum* Group II, while all the other subtypes are solely located in *C. botulinum* Group I. This may indicate that a window of opportunity for horizontal gene transfer of type B toxin genes between the two *C. botulinum* Groups in the distant evolutionary past has now closed. (It is noted, however, that while the *C. botulinum* Group II strain Prevot 59 in our laboratory possessed the type B4 neurotoxin gene, the version of this strain examined by Hill possessed the type B2 neurotoxin gene [75]). The cluster at top left of Fig. 6, which is designated ‘B?’ contains type B genes that are described as type B1 (M-18/3) and type B2 (Osaka06, CDC 6291, 115B), but in this analysis seem to form a separate group (subtype information for strain 115B was derived from microarray data, GEO accession number GSM402938). This appears to be yet another example of *C. botulinum* neurotoxin genes which fail to fit into a nicely defined subtype box.

**Fig. 7:**
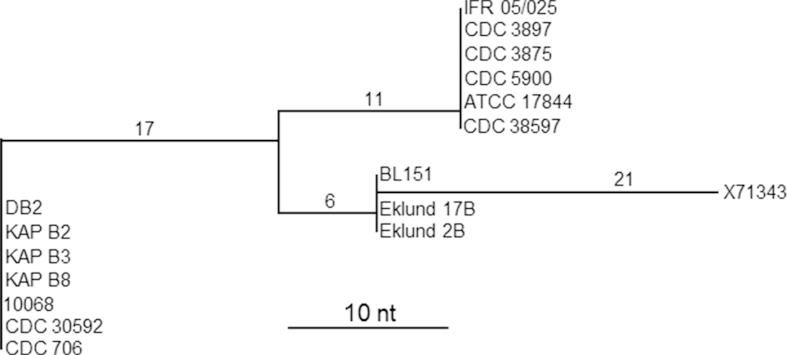
Dendrogram generated by comparison of neurotoxin genes of *C. botulinum* Group II subtype B4 (modified from Ref. [57]). The sequence with accession number X71343 was published as being that of Eklund 17B [77]; however it differs from the two other published versions (which are identical) by 21 nt. Approximate values for the nucleotide differences used to generate the tree branch points are positioned above the major branches.

Analysis of the gene sequence of the neurotoxin subtype B4 reveals further subdivisions (Fig. 7). All of the type B4 genes examined so far are located on an extra-chromosomal element. Recently the sequence of eleven of these, all carried by a plasmid, has been published [57]. Based on this recent work and previous studies, three main sub groups within the type B4 subtype have been identified [57,75,77,78] (Fig. 7). It seems that small genetic differences between the neurotoxin genes, at the SNP level, are not random but become fixed and are associated with a particular subdivision. For strains that formed type A1 neurotoxin, the subdivisions were associated with a specific genetic background (Fig. 5), but this does not seem to be the case for strains that form type B4 neurotoxin [57]. However the three different types of plasmid identified which carry the type B4 neurotoxin gene cluster, do show a link with geographic location [57].

As both the cost and difficulty of bacterial genome sequencing continues to lessen, and the computing power needed to perform analysis of the data subsequently produced becomes more readily available, it can be expected that new *C. botulinum* neurotoxin serotypes and their subtypes will be identified, and that unexpected genome organisations will be uncovered. A perfect example of this was described recently by Raphael and colleagues, who identified the *C. botulinum* Group II type E strain CDC 66177, previously isolated in Argentina in 1995, as not only forming a new neurotoxin subtype (E9), but also enjoying a chromosomal organisation that appears to respect that of the *C. botulinum* Group II type B forming strain, Eklund 17B more than that of published examples of type E strains such as Alaska or Beluga [79].

## Conflict of interest

The authors declare no conflict of interests.

## Figures and Tables

**Table 1:** Characteristics of *Clostridium botulinum* Groups I and II [4–6].

Neurotoxigenic clostridia	*C. botulinum* Group I (proteolytic *C. botulinum*)	*C. botulinum* Group II (non-proteolytic *C. botulinum*)
Neurotoxins formed	A, B, F, H^a^	B, E, F
Ferment glucose	+^b^	+
Ferment maltose	v	+
Ferment fructose	v	+
Ferment sucrose	−	+
Ferment mannose	−	+
Proteolysis^c^	+	−
Liquefaction of gelatin^c^	+	+
Lipase production	+	+
Lecithinase	−	−
Degradation of chitin	+	+
Minimum growth temperature	12 °C	3 °C
Optimum growth temperature	37 °C	30 °C
Minimum pH for growth	4.6	5.0
NaCl concentration preventing growth	10%	5%
Minimum water activity for growth with NaCl	0.94	0.97
Minimum water activity for growth with glycerol	0.93	0.94
Spore heat resistance^d^	*D*_121°C_ = 0.21 min	*D*_82.2°C_ = 2.4/231 min^e^
Non-neurotoxigenic equivalent clostridia	*C. sporogenes*	No species name given

a More than one toxin type may be formed; type H neurotoxin not yet verified, see text.

b + all strains positive, v some strains are positive and others negative, − all strains negative.

c Proteolysis denotes an ability to degrade native proteins (e.g. coagulated egg white, cooked meat particles, casein). Both *C. botulinum* Groups I and II can degrade the derived protein, gelatine.

d Decimal reduction time (*D*-value; i.e. time to a ten-fold reduction in viable spores) at specified temperature determined in phosphate buffer, pH 7.0.

e *D*-value without/with lysozyme during recovery.

**Table 2:** Examples of recent incidents of foodborne botulism.

Outbreak	Probable cause of outbreak	Number of cases (deaths)	*C. botulinum* Group	Toxin type	Reference
Home-made fermented beaver tail and paw (USA, 2001)	Temperature abuse	3 (0)	II	E	[23]
Home-made, fermented salmon roe (2 outbreaks) (Canada, 2001)	Temperature abuse	4 (0)	II	E	[24]
Raw “muktuk” (skin and blubber from beluga whale, stored in sealed plastic bags) (USA, 2002)	Temperature abuse enabling growth and toxin production in sealed bag	12 (0)	II	E	[25]
Home-salted, air-dried fish (Germany, 2003)	Fish gutted, salted in brine, dried, no refrigeration	3 (0)	II	E	[26]
Home-made “rakfisk” (Norway, 2003)	Inadequate salt and inadequate refrigeration	4 (0)	II	E	[26]
Green olives preserved using salt (Italy, 2004)	No lethal heat treatment, no controlled inhibitory conditions	28 (0)	I?	B	[27]
Illicit prison alcohol “pruno”, made with potatoes (2 unlinked outbreaks) (USA, 2004)	Mild heat treatment, maintenance at ambient temperature	5 (0)	I	A	[28]
Home-made suzme (condensed yoghurt) (Turkey, 2005)	Condensed yoghurt filled into plastic jars and buried in earth for 2 months. Yoghurt in one jar had been in contact with soil.	10 (2)	I	A	[29]
Home-dried fish (Kazakhstan, 2005)	Inadequate processing (?)	25 (1)	II?	E?	[30]
Home-prepared, uneviscerated, salted fish (USA, 2005)	Uneviscerated fish with salt placed in sealed plastic bag and stored at ambient temperature for ∼ I month	5 (0)	II	E	[31]
Traditional soup (“Ashmast”) (Iran, 2006)	Soup included spinach that had been stored in an airtight container	11 (0)	II	E	[32]
Home-canned bamboo shoots (Thailand, 2006)	Inadequate heat treatment	209 (0)	I	A	[33,34]
Commercial pasteurised carrot juice (Canada & USA, 2006)	Safety relied on refrigeration; refrigeration inadequate	6 (1)	I	A	[35,36]
Commercially produced sausages (China, 2007)	Production unknown, no refrigeration	66 (0)	I	A	[37]
Commercially canned chilli sauce (USA, 2007)	Deficient canning process	8 (0)	I	A	[38,39]
Home-packed, unprocessed black olives? (Dutch tourists) (Turkey, 2008)	Unknown	8 (0)	I?	B	[40]
Commercial chicken enchiladas (France, 2008)	Product pasteurised, probably stored at room temperature for 2 weeks. Reheated by microwaving	2 (0)	I	A	[41]
Home-canned green beans/carrots (USA, 2008)	Inadequate heat treatment	4 (0)	I	A	[30]
Prepared vegetables (Rwanda, 2009)	Poor preparation (?)	64 (2)	I?	B	[30]
Commercial vacuum-packed, hot-smoked whitefish (France, 2009)	Temperature abuse (?)	3 (0)	II	E	[42]
Commercial products, artichoke preserve; cream of vegetable soup (unlinked cases) (Italy, 2010)	Products pasteurised. Refrigeration not specified or only suggested. Long shelf-life	2 (0)	I?	B	[43]
Home-prepared ham (2 outbreaks) (France, 2010)	Inadequate processing	10 (0)	II	B	[44]
Commercial curry sauce in jar (UK, 2011)	Controlling factor (pH), not as specified	3 (0)	I	A	[45]
Commercial olives stuffed with almonds (from Italy) (Finland, 2011)	Product pasteurised. No inhibitory conditions reported	2 (1)	I	B	[46]
Commercial (artisan) ground green olive paste (France, 2011)	Inadequate heat treatment	9 (0)	I	A	[47]
Commercially produced potato soup (2 unlinked cases) (USA, 2011)	Not refrigerated	2 (0)	I	A	[48]
Illicit prison alcohol “pruno”, made with potatoes (USA, 2011)	Mild heat treatment, maintenance at ambient temperature	8 (0)	I	A	[49]
Home-made olive and tuna pate (?) (Spain, 2011)	Temperature abuse	2 (0)	I	A	[50]
Illicit prison alcohol “pruno”, made with potatoes (2 outbreaks) (USA, 2012)	Mild heat treatment, maintenance at ambient temperature	12 (0)	I	A	[51]
Home-prepared ham (France, 2012)	Inadequate processing	2 (0)	II	B	[44]

**Table 3:** Location and accessory gene status of *C. botulinum* Group I neurotoxin gene clusters.

Neurotoxin subtype	Representative strain	Neurotoxin cluster	Neurotoxin cluster location	Neurotoxin cluster locus	Accession number
A1	ATCC 3502	*ha*	Chromosome	*oppA/brnQ*	AM412317
A1(B)	NCTC 2916	*orf-X, ha*	Chromosome	*arsC* (*oppA/brnQ*)	ABDO02000001-49
A2	Kyoto-F	*orf-X*	Chromosome	*arsC*	CP001581
A2b5	CDC 1436	Unknown	Plasmid	Unknown	[70]
A2f4f5	Af84	*orf-X*	Chromosome	*arsC, pulE*, plasmid-borne site	AOSX00000000.1
			+Plasmid		AOSX01000021.1
A3	Loch Maree	*orf-X*	Plasmid	Plasmid-borne site	CP000962
B5a4	CDC 657	*ha, orf-X*	Plasmid	Plasmid-borne site	CP001081
A5(B′)	H04402 065	*ha*	Chromosome	*oppA/brnQ*	FR773526
B1	Okra	*ha*	Plasmid	Plasmid-borne site	CP000940
B1	CDC 1632	Unknown	Chromosome	Unknown	[70]
B2	CDC 1828	Unknown	Chromosome	Unknown	[70]
B2	ISS-333	Unknown	Plasmid	Unknown	
B3	CDC 816	Unknown	Chromosome	Unknown	[70]
A2b3	ISS-87	Unknown	Plasmid	Unknown	
B5f2	Bf	*ha, orf-X*	Plasmid	Plasmid-borne *orf-X and ha* cluster sites	ABDP010000023,-18,-34, −69 (4 contigs)^a^
F1	Langeland	*orf-X*	Chromosome	*arsC*	CP000728

a Four contigs from the unassembled genome identified as showing regions of homology to other neurotoxin cluster-bearing plasmids were assembled manually [69]. Ref. [70], a PFGE analysis, has been cited where more specific information is unavailable.

**Table 4:** Location and accessory gene status of *C. botulinum* Group II neurotoxin gene clusters.

Neurotoxin subtype	Representative strain	Neurotoxin cluster	Neurotoxin cluster location	Neurotoxin cluster locus	Accession number
B4	Eklund 17B	*ha*	Plasmid	Plasmid-borne site^a^	FR745876, CP001057
E1	Beluga	*orf-X*	Chromosome	*rarA*	ACSC000000001-16
E1	CB11/1-1	*orf-X*	Plasmid	Plasmid-borne site	AORM01000001-171
E3	Alaska E43	*orf-X*	Chromosome	*rarA*	CP001078
E9	CDC 66177	*orf-X*	Chromosome	*rarA*	ALYJ01000001-119
F6	IFR 06/001	*orf-X*	Chromosome	*topB*	KC516868^b^

Strain Eklund 17B has been independently sequenced by two institutes; the two accession numbers are for both versions of the neurotoxin gene cluster-bearing plasmid. Note: no genome sequence data are available for subtypes E2, E6, E7 and E8. Subtypes E4 and E5 are found exclusively in strains of neurotoxigenic *C. butyricum.*

a The neurotoxin gene cluster-bearing plasmid of *C. botulinum* Group II type B4 does not display synteny with those of *C. botulinum.* Group I

b Sequence of a 50 kb region of the chromosome containing the neurotoxin gene cluster.
